# Plan meta-objective for sub-micron quantitative phase imaging

**DOI:** 10.1038/s41377-025-02099-z

**Published:** 2026-01-20

**Authors:** Junyi Wang, Jiacheng Sun, Jian Li, Chunyu Huang, Jitao Ji, Wenjing Shen, Zhizhang Wang, Junxiao Zhou, Chen Chen, Shining Zhu, Tao Li

**Affiliations:** National Laboratory of Solid State Microstructures, Key Laboratory of Intelligent Optical Sensing and Manipulations, College of Engineering and Applied Sciences, Jiangsu Key Laboratory of Artificial Functional Materials, and Collaborative Innovation Center of Advanced Microstructures, Nanjing University, Nanjing, China

**Keywords:** Imaging and sensing, Phase-contrast microscopy, Metamaterials

## Abstract

Quantitative phase imaging (QPI) provides valuable objective insights for investigating transparent samples, yet miniaturizing QPI systems without compromising performance remains a critical challenge for applications requiring compactness and portability. Here, by introducing partially coherent illumination modulation, together with a plan meta-objective (PMO) design, we present a compact QPI system with sub-micron resolution. The PMO is a monolithically integrated doublet metalens with its dispersion enabling focal shifts at two wavelengths, obviating the need for mechanical translations during image acquisition for phase retrieval. The PMO is also optimized to correct for monochromatic aberrations, delivering an object-side field of view equivalent to ~90% of the lens aperture with minimal distortion and aberrations. The spatial coherence of the illumination is controlled to enhance imaging resolution. By co-designing illumination and imaging systems, we demonstrate QPI achieving a half-pitch lateral resolution of 488 nm with a phase accuracy of 0.06λ. Our approach enables high-quality QPI analysis of diverse phase objects, including unstained biospecimens, laying the foundation for the development of compact, stable, and practical QPI platforms.

## Introduction

Quantitative phase imaging (QPI) techniques^1–7^ leverage the phase information of optical fields as an endogenous contrast mechanism to visualize the morphology of transparent samples. The quantitative data obtained through QPI, which encodes the refractive index and thickness of the sample, enables precise cell mass measurement^8,9^, and reduces subjectivity in disease diagnosis, thereby advancing digital pathology and automated cell analysis^10–12^. Furthermore, as a label-free approach, QPI significantly accelerates current clinical diagnostic workflows, while mitigating challenges such as photobleaching and phototoxicity during long-term cell observations^13^. However, conventional QPI techniques rely on bulky optical components, leading to complex and cumbersome system configurations. This limitation hinders their applicability in scenarios requiring compact and portable solutions, such as point-of-care diagnostics and in vivo applications.

Metasurfaces and metalenses, two-dimensional planar optical elements composed of subwavelength artificial meta-atoms^14,15^, have demonstrated great potential for miniaturizing imaging systems^16–24^. Their multifunctional design flexibility has driven notable advancements in compact QPI platforms, yet critical limitations persist. Interferometric metasurface-based QPI methods^25^ offer high precision but face environmental sensitivity and limited resolution. Though common-path configurations can mitigate these issues, they introduce increased complexity in image acquisition^26–29^. Alternatively, the transport of intensity equation (TIE) offers a convenient phase retrieval method with only axially displaced images. Metasurface-based TIE techniques^30–35^ eliminate the need for mechanical scanning, improving the system stability and acquisition speed, albeit at the expense of image definition or sensor size. Despite the progress, current metasurface-based QPI works remain proof-of-concept demonstrations, still fall short of standards for practical applications, particularly in terms of lateral resolution and object-side field of view (the object-side field of view is hereinafter abbreviated as FoV). Two fundamental factors underlie the challenge: first, singlet metalenses suffer from severe off-axis aberrations that worsen with increasing numerical aperture (*NA*), reducing phase accuracy and resolution; second, the phase retrieval methods employed are constrained by the paraxial approximation or the need for high spatial coherence, thereby limiting the lateral resolution^36–38^.

**Fig. 1:**
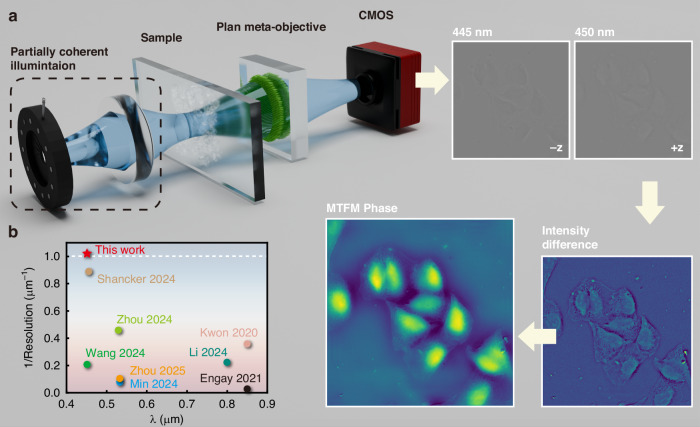
Schematics of the QPI method based on a plan meta-objective and resolution comparison with the state of the art. **a** PCI is obtained by placing an aperture diaphragm at the front focal plane of the condenser lens to improve imaging resolution. The plan meta-objective is a monolithically integrated doublet metalens optimized to provide two aberration-free and near-symmetrically defocused images separated by a small distance under different illumination wavelengths (445 nm and 450 nm), without translating the object or sensor. MTFM is employed for a high-resolution and high-accuracy phase retrieval. **b** Comparison of the resolution of the proposed method and the state of the art. The y axis represents the reciprocal of the imaging resolution (full-pitch resolution), and the white dashed line marks the boundary of sub-micron full-pitch resolution

In this study, we proposed and experimentally demonstrated a compact QPI system capable of sub-micron phase reconstruction using a plan meta-objective (PMO) under partially coherent illumination. The PMO was optimized through ray tracing to provide a distortion- and aberration-free FoV, together with its natural dispersion enabling non-mechanical object plane shifts at two working wavelengths (445 nm and 450 nm). By positioning the sample between the designed object planes, two near-symmetrically defocused images were captured under corresponding wavelengths without sacrificing the sensor size or image definition. The mixed transfer function method (MTFM)^39^ was employed to achieve QPI with a half-pitch resolution of 488 nm (highest resolution compared to the state of the art, see Fig. 1b and Supplementary Table S1). The phase retrieval accuracy was validated using a commercial microlens array, demonstrating a root mean square error (RMSE) of 0.06λ compared to the surface profile measured by an atomic force microscope (AFM). Additionally, we demonstrated the QPI performance on a phase resolution test chart and unstained cells cultured in vitro, revealing surface morphology with high resolution and accuracy.

## Results

### Principle

Figure 1a illustrates the concept of the proposed QPI method, which combines a partially coherent illumination (PCI) configuration with a PMO-based imaging system. The PCI can extend imaging resolution beyond the coherent diffraction limit^37,38,40^. The doublet is optimized to minimize monochromatic aberrations at two working wavelengths (${\lambda }_{1}=445nm$ and ${\lambda }_{2}=450nm$), and its natural dispersion at these wavelengths can provide object plane shifts while maintaining a fixed image plane. Two near-symmetrically defocused images (${I}_{{\lambda }_{1,2}}$) can be captured by adjusting the illumination wavelength to calculate an intensity difference for phase retrieval. The in-focus phase distribution is first derived from the TIE using the intensity difference as

1 $${\tilde{\varphi }}_{TIE}=\frac{{\tilde{I}}_{{\lambda }_{2}}-{\tilde{I}}_{{\lambda }_{1}}}{{I}_{0}{{H}}_{TIE}}$$

where ${\tilde{\varphi }}_{TIE}$ is the Fourier transform of the phase recovered using TIE, ${\tilde{I}}_{{\lambda }_{1,2}}$ are the Fourier transform of two defocused images, ${{H}}_{TIE}$ is the phase transfer function of the TIE (see Supplementary Note 1), and ${I}_{0}$ is a constant. Due to the limited objective pupil and the paraxial approximation, the imaging point spread function will severely reduce the contrast of high spatial frequency phase information under PCI^36^. Therefore, the TIE can only accurately recover a slowly varying phase envelope of the optical field. To recover high-frequency phase information under PCI, the weak object transfer function (WOTF) is subsequently employed, and the phase is given by

2 $${\tilde{\varphi }}_{WOTF}=\frac{{\tilde{I}}_{{\lambda }_{2}}-{\tilde{I}}_{{\lambda }_{1}}}{2{H}_{WOTF}}$$

where ${\tilde{\varphi }}_{WOTF}$ is the Fourier transform of the phase recovered using WOTF, ${H}_{WOTF}$ is the phase transfer function of the WOTF (see Supplementary Note 1). The WOTF is valid under weak object approximation, which the strict definition gives the object phase as no larger than 0.5 rad^41^. When samples do not fall within the category, nonlinear errors degrade the phase reconstruction accuracy.

By employing the MTFM^39^, which combines the accurate phase envelope derived from the TIE with the high-spatial-frequency information recovered by the WOTF in the spatial frequency domain, a high-resolution and high-accuracy phase image can be retrieved as

3 $$\varphi ={F}^{-1}\{LP\cdot {\tilde{\varphi }}_{TIE}+HP\cdot {\tilde{\varphi }}_{WOTF}\}$$

where $\varphi$ is the reconstructed phase, *LP* (*HP*) is a low (high) pass filter in the spatial frequency domain.

### Design and characterization

**Fig. 2:**
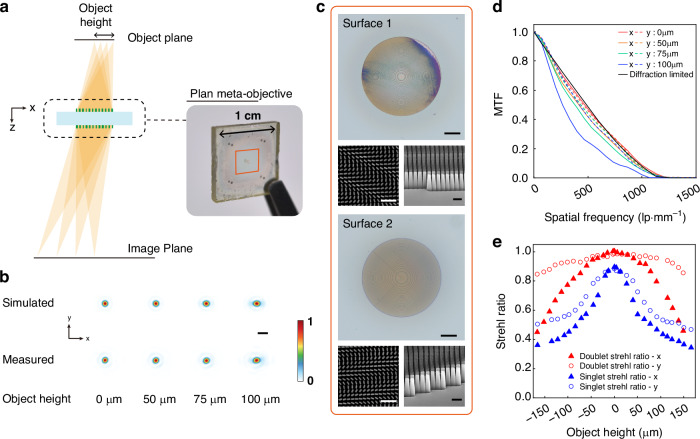
Characterization of the fabricated PMO. **a** The PMO is optimized using ray tracing to transfer objects from different object heights to diffraction-limited images with a specific magnification. A photograph of the PMO is shown in the inset. **b** Comparison between the simulated and measured PSFs of the PMO with a point source located at different object heights. Scale bar: 2 μm. **c** Optical images of the two surfaces (scale bars: 100 μm) and SEM images of the nanofins on respective surfaces **(**scale bars: 1 μm for top views and 500 nm for side views). **d** MTFs along x and y directions calculated from the measured PSFs in **b**. **e** Strehl Ratios of the PMO and a singlet metalens across a FoV of 320 μm

Uncontrolled optical aberrations, other than defocus, degrade the phase reconstruction accuracy and introduce undesired phase artifacts. Singlet metalenses suffer from severe off-axis aberrations, leading to severe distortion and blurring of images. To address these issues, a doublet design was employed to offer an extra design flexibility for the ray tracing algorithm to reduce the monochromatic aberrations and provide uniform focusing capability across the image field^42,43^. The ray tracing illustration is shown in Fig. 2a, with a photograph of the fabricated PMO shown in the inset. The PMO is a monolithically integrated doublet metalens. This double-sided design ensures compactness and mechanical robustness and obviates sophisticated alignment during experiments. Optical and scanning electron microscopy (SEM) images of the two surfaces are displayed in Fig. 2c. The metalenses on both surfaces have the same diameter of 500 μm. Surface 2 serves as the aperture stop, which limits the doublet’s *NA* to 0.27 at the object space. The SiO_2_ spacer between the two metalenses is 1.2 mm in thickness with a refractive index of 1.47. The imaging magnification is designed to be 4 at the working wavelength of 450 nm and 3.977 at 445 nm, with a 10-μm shift in the object plane. The total track length of the design is less than 5.6 mm. The optimization was performed using Ansys Zemax OpticStudio, and the phase profile follows the expression as $\varphi (r)=\mathop{\sum }\limits_{n=1}^{7}{a}_{n}{r}^{2n}$ (see Supplementary Table S2 for the coefficients ${a}_{n}$). The phase profiles of the two metalenses are designed using the Pancharatnam-Berry phase method via distributing SiN_x_ nanofins (height is 1000 nm, length is 210 nm, width is 72 nm) in a rectangle lattice with a 260-nm period (see Materials and Methods for simulation and fabrication details).

The point spread function (PSF) and modulation transfer function (MTF) of the PMO were first characterized using a custom-built optical configuration (see Materials and Methods and Supplementary Fig. S1). The radius of the near-diffraction-limited FoV is defined as the object height at which the Strehl ratio exceeds 0.8 (the Strehl ratio is defined as the ratio of the area under the MTF curve to that under the diffraction-limited MTF curve). As shown in Fig. 2b, the measured PSFs at a wavelength of 450 nm for four object heights along the x direction closely match the simulated PSFs and demonstrate an expansion along the x direction with the increasing object height (see Materials and Methods for the diffraction simulation details, the simulated PSFs at a wavelength of 445 nm are shown in Supplementary Fig. S2, and the optical layouts and simulated spot diagrams in Zemax are presented in Supplementary Fig. S3). The slight tilting of the measured PSFs is attributed to misalignment in the experiment. The MTFs and the Strehl ratios of the doublet are shown in Fig. 2d, e, validating the near-diffraction-limited performance across the FoV of 200 μm along x and y directions (the comparisons between the measured MTFs and Strehl ratios and those from Zemax simulation are shown in Supplementary Fig. S4). Figure 2e also compares the measured Strehl ratios between the PMO and a singlet metalens with similar *NA* across a larger FoV of 320 μm. The singlet metalens follows an on-axis spherical-aberration-free phase profile (see Supplementary Note 2). The singlet metalens has a near-diffraction-limited FoV of only 80 μm, and its Strehl ratio rapidly drops outside the central field. In contrast, the Strehl ratios of the PMO remain above 0.8 in the y direction and are higher than those of the singlet metalens in both directions. These results indicate that the PMO offers a more uniform and larger FoV compared to the singlet metalens.

**Fig. 3:**
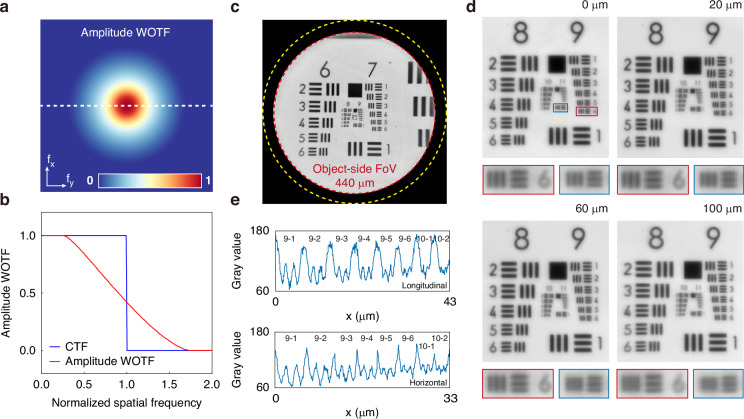
Intensity imaging performance of the PMO. **a** Illustration of the amplitude WOTF at a wavelength of 450 nm in the spatial frequency domain. **b** Comparison between the CTF (blue line) and the amplitude WOTF (red line) along the white dashed line in **a**. **c** Full-field image of the doublet with a diameter of 440 μm at a wavelength of 450 nm after flat-field correction. The yellow dashed circle represents the 500-μm lens aperture. The red one indicates the 440-μm FoV. **d** Images of the central region after the relay system at object heights of 0 μm, 20 μm, 60 μm, and 100 μm along the horizontal direction. The magnified images of 9-6 and 10-1 line pairs are displayed below the corresponding images. **e** Longitudinal and horizontal intensity profiles of the elements in group 9, as well as the 10-1 and 10-2 line pairs at an object height of 0 μm, demonstrating isotropic resolving power

The intensity imaging performance of the PMO was characterized under PCI using a customized positive resolution test chart (see Materials and Methods) at the working wavelength of 450 nm. The coherent parameter is defined as $S=N{A}_{illum}/N{A}_{lens}$. The resolution of the imaging system can be approximated with $\lambda /(1+S)N{A}_{lens}$, where *S* = 0 corresponds to a spatially coherent imaging system, and *S* = 1 corresponds to a spatially incoherent system^36^. In the following imaging test, *S* was set as 0.74, meaning $N{A}_{illum}=0.2$ and the ideal half-pitch resolution was 479 nm at a wavelength of 450 nm. The amplitude transfer function, described by the WOTF (amplitude WOTF) was calculated at a wavelength of 450 nm (Fig. 3a), and the profile along the white dashed cutline is compared with the CTF (Fig. 3b). The amplitude WOTF shows an extended cut-off frequency due to PCI, indicating an improvement in lateral resolution. The full-field image, after flat-field correction (see Materials and Methods), was captured directly by a commercial imaging sensor (Imaging source: DMM 27UJ003-ML, pixel size: 1.67 × 1.67 μm) at a wavelength of 450 nm (Fig. 3c). The PMO demonstrated an FoV of 440 μm with minimal distortion and blurring (i.e., an effective FoV 88% of the lens aperture) compared to the image formed by the singlet metalens (Supplementary Fig. S5). The image field beyond this region was blurred due to decreasing focusing efficiency and was therefore cropped. With a magnification of 4, the effective pixel size can satisfy the Nyquist sampling criterion. However, unavoidable pixel noise (thermal noise, dark current noise, etc.) degraded the imaging resolution to element 4 in group 9 (see Supplementary Fig. S7 for more imaging results). Oversampling is required to ensure that the PMO’s resolution capability remains uncompromised. A custom-built microscope (see Materials and Methods and Supplementary Fig. S1) with a magnification of 10 was introduced after the doublet (see the complete image field after the relay system in Supplementary Fig. S8). The images of the center area of the resolution test chart after relaying are shown in Fig. 3d. To assess the PMO’s resolving capability, element 1 in group 10 (10-1 line pairs, corresponding to a 976-nm full-pitch resolution or 488-nm half-pitch resolution) was precisely positioned at the center FoV. The resolution test chart was then translated along the horizontal direction, and images were captured at four selected positions. The relative object height was determined based on the pixel displacement of the 10-1 line pairs in each image. As shown in the magnified images of 9-6 and 10-1 line pairs in Fig. 3d, contrast gradually decreases with increasing object height, agreeing well with the design expectations. The horizontal and longitudinal intensity profiles of the elements in group 9, as well as the 10-1 and 10-2 line pairs at object height of 0 μm demonstrate that 10-1 line pairs remain distinguished, validating the PMO’s diffraction-limited performance on axis (Fig. 3e).

### QPI performance

**Fig. 4:**
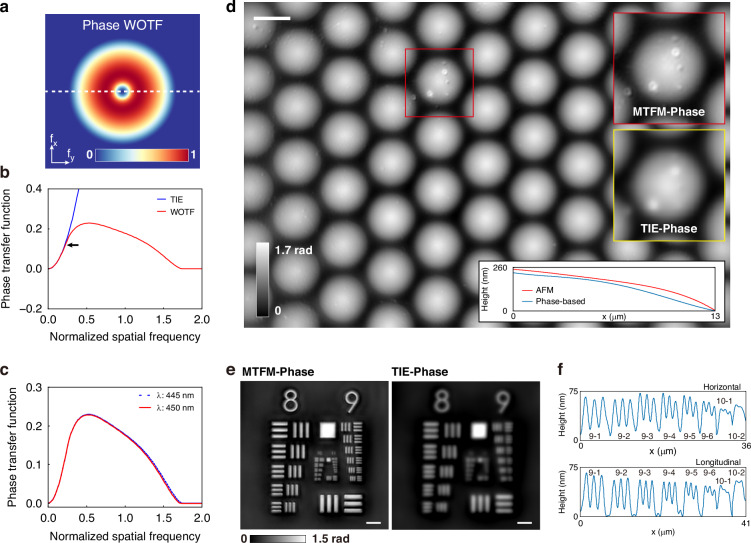
Characterization of the phase recovery accuracy and resolution. **a** The phase WOTF calculated from the PCI model at a wavelength of 450 nm in the spatial frequency domain. **b** Comparison between the phase transfer function of the TIE (blue line) and the phase WOTF along the white dashed line in **a** (red line). The black arrow indicates the position where the difference between the two functions is approximately 0.01. **c** Comparison of the horizontal cutlines of the phase WOTF at wavelengths of 445 nm and 450 nm. **d** Reconstructed phase profile of the microlens array. The right inset compares the phase in the red box region recovered by the MTFM and the TIE. The bottom inset compares the radially averaged height of the microlens array derived from the phase with the height measured by AFM. Scale bar: 20 μm. **e** Reconstructed profiles of the phase resolution test chart using the MTFM and the TIE. Scale bars: 8 μm. **f** Surface profiles of elements in group 9, as well as the 10-1 and 10-2 line pairs along the horizontal and longitudinal directions

A commercially available fused-silica microlens array with a hexagonal lattice (refractive index is 1.47 and lattice period is ~26 μm) was used to evaluate the phase reconstruction accuracy of the QPI method based on the PMO. The coherent parameter was set to *S* = 0.74, consistent with the intensity imaging tests described above. Figure 4a presents the phase transfer function described by the WOTF (phase WOTF), which shares the same cutoff spatial frequency as the amplitude WOTF ($1.74N{A}_{lens}/\lambda$). The profile along the white dashed line is shown as the red line in Fig. 4b. The phase transfer function of the TIE coincides well with the phase WOTF around the low-frequency region, indicating the slowly varying phase envelope retrieved by the TIE is valid under PCI (Fig. 4b). By combining the high-spatial-frequency information recovered by the WOTF with the accurate phase envelope provided by the TIE, a high-resolution and high-accuracy phase image was reconstructed. The frequency boundary is indicated by a black arrow in Fig. 4b, where the difference between the two functions is 0.01. The phase WOTFs at the wavelengths of 445 nm and 450 nm display negligible discrepancies (Fig. 4c), validating the feasibility of our dispersion-assisted phase reconstruction method, with 450 nm chosen as the center wavelength for reconstruction. By placing different 10-nm bandwidth filters with center wavelengths of 445 nm and 450 nm, respectively, after the illumination source, two near-symmetrically defocused images were obtained without requiring mechanical translation of the object or image plane. The reconstructed phase distribution of the microlens array, derived using the MTFM, displays identical round shapes (Fig. 4d). The phase distribution can be transformed into a height (*h*) profile using the relation $h=\lambda \varphi /2\pi \varDelta n$, where $\varDelta n$ is the refractive index difference between the microlens array and the ambient medium. The region enclosed by a red box was selected to compare the MTFM-based and TIE-based phase images, as shown in the inset in Fig. 4d, where the MTFM-based phase image demonstrates finer structure details. To evaluate the accuracy of our QPI method, the radially averaged height measured by AFM was used as the ground truth. The phase-based surface profile was obtained by averaging the radially averaged heights of different sub-micolenses within the near-diffraction-limited FoV. The comparison is shown at the bottom of Fig. 4d. The RMSE between the two profiles is 27 nm, corresponding to 0.06λ.

We further assessed the phase reconstruction resolution using a phase resolution test chart composed of SiN_x_ (see Materials and Methods). The thickness of the test chart was measured as 100 ± 10 nm using a stylus profilometer (KLA, Tencor P-7). Compared to the phase image retrieved by the TIE, that retrieved by the MTFM exhibits a significant improvement in imaging resolution (Fig. 4e). The MTFM-based surface profiles (Fig. 4f) reveal that the reconstructed heights are about 70 nm, showing an underestimation of approximately 30 nm, which aligns with the expected accuracy of our method. As the features approach the diffraction limit, the contrast of the recovered profiles gradually diminishes. The 10-1 line pairs remain distinguished, whereas the 10-2 line pairs become indiscernible, indicating that the half-pitch resolution of the phase image is 488 nm, consistent with the theoretical limit.

**Fig. 5:**
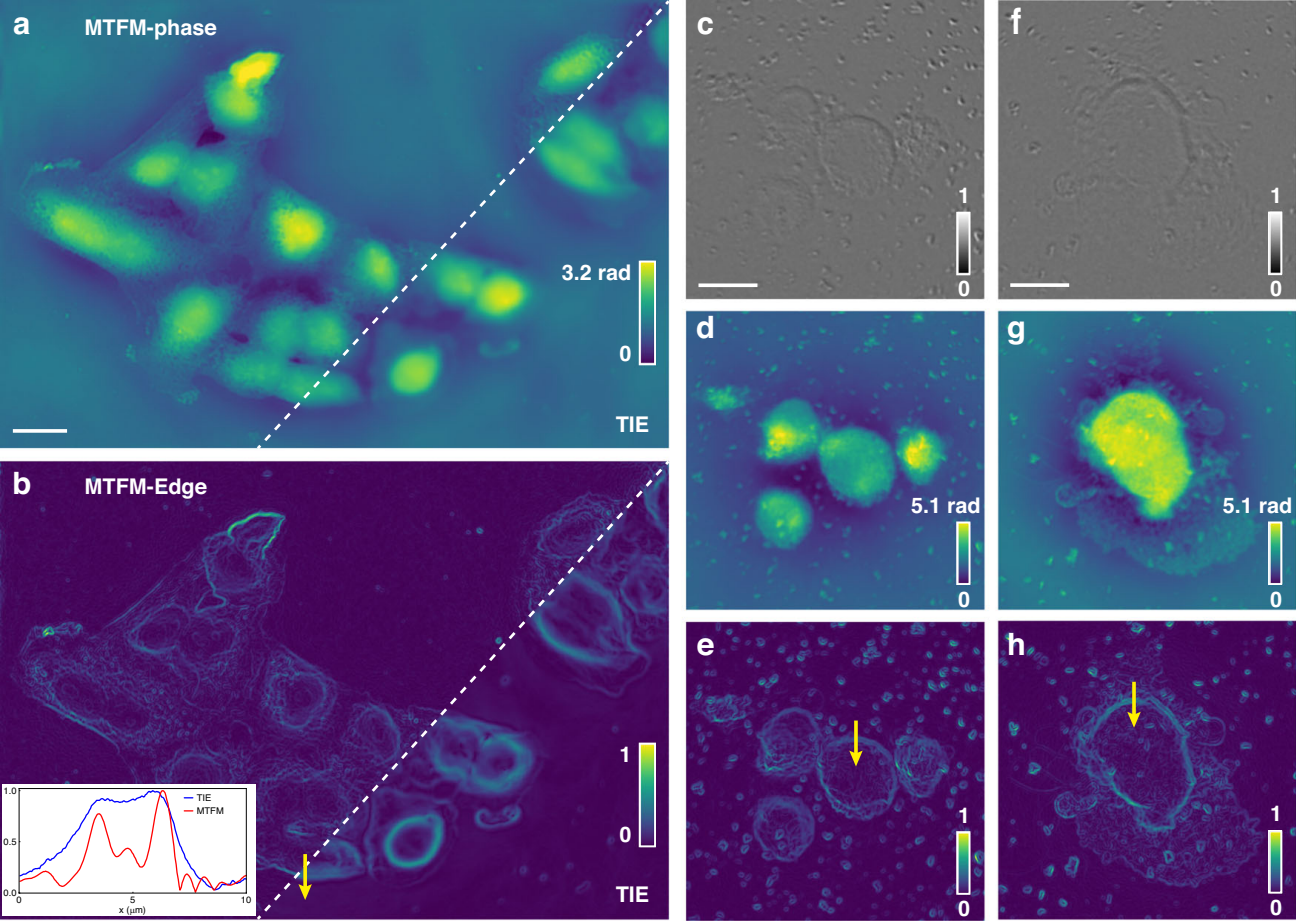
Quantitative phase imaging of unstained biospecimens. **a** Comparison of phase images of HeLa cells reconstructed by the MTFM and TIE. Scale bar: 20 μm. **b** Comparison of edge information extracted from the phase images. The inset compares the edge profiles along the yellow arrow. **c** Intensity and **d** phase images of the digested primary human cervical epithelial cells. Scale bar: 20 μm. **e** Edge information extracted from **d**. **f** Intensity and **g** phase images of the digested human cervical cancer cell. Scale bar: 20 μm. **h** Edge information of **g**. The edge profiles along the yellow arrow are shown in Supplementary Fig. S10

After validating the accuracy and resolution of the method, we further tested its performance on two types of unstained biospecimens cultured in vitro to showcase its potential applications. Figure 5a displays the reconstructed phase image of a HeLa cell slide (see Supplementary Fig. S9 for the in-focus intensity image). Compared to the phase retrieved by the TIE, the MTFM-based phase image reveals finer details of the cell morphology, and edge information can be directly extracted with higher precision (Fig. 5b). Due to the enhanced resolution, the MTFM-based edge profile along the yellow arrow demonstrates sharper edges compared to the TIE-based edge profile (see the inset in Fig. 5b). The two obvious edges correspond to the nucleus-cytoplasm boundary and the interface between the cytoplasm and the ambient medium. The improved edge resolution can enhance the robustness of the automated analyses, such as cell segmentation. The in-focus intensity, phase, and edge images of two types of digested cells are demonstrated in Fig. 5c–e and 5f–h (Fig. 5c–e correspond to primary human cervical epithelial cells, and Fig. 5f–h depict a primary human cervical cancer cell). The phase images showcase more detailed cell morphology compared to near-unity intensity images. The edge profiles along the yellow arrows in Figs. 5e and h are shown in Supplementary Fig. S10, also showcasing an obviously improved edge resolution compared to the TIE-based edge profiles. Furthermore, by comparing the results in Fig. 5d, g, the primary cervical cells cultured in vitro exhibit a large nuclear-cytoplasmic ratio, and the cancer cell is much larger than the normal cells in size and optical thickness. These insights, which are difficult to extract from intensity images, can be quantitatively assessed using the phase information. By analyzing parameters such as dry mass, nuclear-cytoplasmic ratio, and cell size from a large sample set, potential diagnostic thresholds can be established to differentiate between normal and cancerous cervical cells, thereby accelerating the diagnostic process in cytopathology.

## Discussion

In conclusion, this work demonstrated a significant advancement in metasurface-based QPI by combining partially coherent illumination with a plan meta-objective, enabling high-quality QPI with a compact and stable system. By co-designing the illumination and imaging systems, as well as employing the MTFM for phase retrieval, we achieved sub-micron resolution and a wide effective FoV. The use of partially coherent illumination and doublet design effectively addresses the tradeoff between FoV and resolution commonly encountered in singlet metalenses. Furthermore, the focal shifting enabled by natural dispersion substantially enhances the acquisition speed and stability of the QPI system. We anticipate that the current system can benefit from further engineering optimizations, such as integrating an image sensor with a smaller pixel size, optimizing imaging magnification (a feasible design of a meta-objective with an imaging magnification of 10 and an *NA* of 0.3 is demonstrated in the Supplementary Note 3 and Fig. S11 to show the potential for a more compact system), synchronizing the filter switching with image acquisition using motorized rotation stage to improve the system’s temporal resolution and employing longer illumination wavelengths to avoid possible harm to cellular physiology, to facilitate broader adoption in diverse practical applications, including cell culture, digital pathology, point-of-care diagnostics, and industrial metrology.

## Materials and methods

### Numerical simulations

We conducted the full-wave simulations using a commercial finite-difference time-domain solver (Lumerical, Ansys). The refractive indices of SiN_x_ are 2.0709 and 2.0673 at *λ* = 445 nm and 450 nm. The refractive indices of SiO_2_ are 1.4724 and 1.4720, respectively. The wavelength-dependent refractive indices were measured using a spectroscopic ellipsometer (J.A.Woollam, RC2). Periodic boundary condition was used in the simulation for calculating the polarization conversion ratio. The SiN_x_ nanofin was set as 1000 nm tall with a rectangle lattice period of 260 nm. The suitable length and width of the meta-atom possessing a high polarization conversion ratio were selected using the particle swarm algorithm.

Diffraction simulations were based on Rayleigh-Sommerfield theory, and the electric field at any position beyond the evanescent region can be expressed as

$$u(x,y,z)=-\frac{1}{2\pi }\int {\int }_{-\infty }^{+\infty }U({x}^{\mathrm{'}},{y}^{\mathrm{'}})g(x-{x}^{\mathrm{'}},y-{y}^{\mathrm{'}},z,\lambda )d{x}^{\mathrm{'}}d{y}^{\mathrm{'}}$$

where $g(\xi ,\eta ,z,\lambda )={e}^{jknR}(ikn-1/R)z/{R}^{2}$, $R=\sqrt{{\xi }^{2}+{\eta }^{2}+{z}^{2}}$, $k=2\pi /\lambda$, and *n* is the refractive index of the ambient medium.

### Device fabrication

The fabrication procedure for the PMO is as follows. A pair of alignment marks was first patterned and aligned on both sides of the 1.2-mm-thick substrate using optical lithography (SUSS MA6BA6). Then, the 1000-nm-thick SiNx layer was deposited on both sides of the substrate, respectively, using plasma-enhanced chemical vapor deposition. A positive electron beam resist (200 nm, PMMA A4) was first spin-coated onto surface 2 and baked at 170 °C for five minutes. Next, a 100-nm-thick layer of a water-soluble conductive polymer (AR-PC 5090) was spin-coated on the resist for the dissipation of electron beam charges. The metasurface pattern was written on the resist using electron beam lithography (EBL, ELS-F125, Elionix). The conductive polymer was then dissolved in water, and the resist was developed in a resist developer solution. Then, the pattern was transferred into a 40-nm-thick Cr layer deposited by electron beam evaporation using the lift-off technique. The patterning of the Cr mask of surface 1 follows the same procedure. After completing the patterning of Cr masks, the device was transferred to Oxford Instruments, PlasmaPro100 Cobra300, and etched with a mixture of CHF3 and SF6 plasma. The flow rate of the two gases was 60 sccm: 6 sccm. Finally, the Cr masks were removed using a solution of ammonium cerium nitrate.

The fabrication of the SiNx phase resolution test chart and the positive resolution test chart follows the same EBL patterning procedure. The patterns were all prepared on 500-μm-thick fused silica substrates. The pattern of the phase resolution test chart was transferred to a 100-nm-thick SiNx layer using the same lift-off and etching procedures. The pattern of the positive resolution test chart was transferred to chromium-gold-chromium layers (the thicknesses are 10 nm, 60 nm, and 20 nm) by an e-beam evaporator (SKY, DZ450), followed by a lift-off process to obtain the designed pattern. The sizes of the line pairs are all set to match the standards of the 1951 USAF resolution test chart.

### Flat-field correction

Flat-field correction can reduce the influence of the vignetting effect or unwanted background signals and create a near-flat-field image. The process first captures a clean background image without the object (${I}_{b}$) and then records the object image (${I}_{o}$). If the exposure time is long, a dark field image is also needed (${I}_{d}$). The final image is given as ${I}_{f}=({I}_{o}-{I}_{d})/({I}_{b}-{I}_{d})$.

### Experimental setup

The imaging performance of the metalens was characterized using the setups shown in Supplementary Fig. S1a. A blue LED (SOLIS-445C, Thorlabs Inc.) was chosen as the light source. An achromatic lens (OLD3254-T2M, Jcoptix) was used to collect the light and image the LED pattern on the aperture diaphragm (AD, MIS1-25, Jcoptix). A field diaphragm (FD, MIS1-25, Jcoptix) was put right after the lens to control the beam size. Filters of 10 nm bandwidth and a left circularly polarized (LCP) film (#88-084, Edmund Optics) were placed between the AD and FD to modulate the illumination. After the AD, an aspherical lens (OLS240417-T2, Jcoptix) was employed as the condenser lens to focus the light and image the FD on the object plane. The two lens systems together formed a Köhler illumination system to provide uniform illumination and controlled spatial coherence. The meta-objective and the samples were mounted on separate translation stages after the condenser lens. The images formed by the meta-objective were relayed to a CMOS image sensor (AIC-2000C-USB, pixel size: 2.4 μm × 2.4 μm, Jcoptix) by a custom-built microscope consisting of an objective lens (NA0.28, 10X, Mitutoyo) and a tube lens (TTL200-A, Thorlabs Inc.). An LCP film was inserted between the objective lens and the tube lens to filter the unwanted signals.

The schematic of the optical configuration for PSF characterization is shown in Supplementary Fig. S1b. A laser (LDM10-450, 10 mW, center wavelength 450 nm, Jcoptix) was used for incidence. An objective lens (20X, NA0.4, GCO-2132, Daheng Optics Co. Ltd.) was combined with a 15 μm pinhole to form a spatial filter. The spatial filter was inserted after the source to create a clean, divergent optical field. The divergent beam was first collimated by an achromatic lens (OLD2440-T2M, Jcoptix) and then focused by an objective lens (LMPLFLN 100X, NA0.8, Olympus) to create a point source. The meta-objective was mounted on a translation stage after the point source. A custom-built microscope consisting of an objective lens (CFI Plan Fluor 40X, NA0.75, Nikon) and a tube lens (TTL200-A, Thorlabs Inc.) was used to relay the light focused by the doublet to a CMOS image sensor. A 50-50 beam splitter was inserted before the image sensor to divide the beam into a power meter (PM120D, Thorlabs Inc.). LCP films were inserted after the source to create a polarized incidence and between the objective lens and the tube lens to filter the unwanted signals.

## Supplementary information

Supplementary information to Plan meta-objective for sub-micron quantitative phase imaging

## Data Availability

The datasets used and analysed during the current study are available from the corresponding author on reasonable request.
