# Supplementary material for: Plan meta-objective for sub-micron quantitative phase imaging
Source: Light Sci Appl. 2026 Jan 20;15:71. doi: 10.1038/s41377-025-02099-z (PMC12816609; doi:10.1038/s41377-025-02099-z)
Supplement: Supplementary file 1 — Supplementary information to Plan meta-objective for sub-micron quantitative phase imaging [file 41377_2025_2099_MOESM1_ESM.docx]

**Supporting Information for**

**Plan meta-objective for sub-micron quantitative phase imaging**

Junyi Wang, Jiacheng Sun, Jian Li, Chunyu Huang, Jitao Ji, Wenjing Shen, Zhizhang Wang, Junxiao Zhou, Chen Chen*, Shining Zhu and Tao Li*

*National Laboratory of Solid State Microstructures, Key Laboratory of Intelligent Optical Sensing and Manipulations, College of Engineering and Applied Sciences, and Jiangsu Key Laboratory of Artificial Functional Materials, Nanjing University, Nanjing, 210093, China.*

*Corresponding author: Chen Chen [(chenchen2021@nju.edu.cn)](mailto:(chenchen2021@nju.edu.cn)) or Tao Li [(taoli@nju.edu.cn)](mailto:(taoli@nju.edu.cn))

**Supplementary Note 1. Principle of the transport-of-intensity equation and the weak object transfer function**

The transport-of-intensity equation (TIE) is written as^1^

(S1)

where $k=2\pi/\lambda$ is the wavenumber, $\lambda$ is the wavelength, *z* is the coordinate along the optical axis, and $x$ is the two-dimensional (2D) spatial coordinate, *I* and represent the intensity and phase distributions at the recorded plane, respectively, and is the 2D gradient operator. Under certain boundary conditions, the absolute phase can be deterministically retrieved without phase unwrapping or iterative approximations. In the wave propagation equation, the wavelength and propagation distance are equivalent, allowing the product of wavelength and defocus distance to be defined as a general defocus distance $\xi=\lambda z$. The TIE solving process is similar. In the simplest form, the axial intensity derivative can be approximated by the intensity difference between two images captured at slightly different phases as

$\frac{\partial I}{\partial\xi}\approx\frac{\left(I_{\xi}-I_{-\xi}\right)}{2\xi}$ (S2)

Since phase objects typically display nearly uniform in-focus intensity distributions, the in-focus intensity can be approximated with a constant $I_{0}$. Under this assumption, substituting Eq. S2 into Eq. S1, Eq. S1 can be simplified as

(S3)

By transforming the Eq. S3 into the spatial frequency domain, a linear relationship between the phase and the axial intensity difference can be established as

(S4)

where $\tilde{I}$ and denote the Fourier transform of the intensity and phase, respectively, and $u$ is the 2D spatial frequency coordinate. The in-focus phase distribution derived from Eq. S4 can be expressed as

(S5)

where $H_{TIE}=4\xi\pi\left|u\right|^{2}$.

An alternative phase retrieval method is the weak object transfer function (WOTF), which is applicable under partially coherent illumination. The image of an object under partially coherent illumination after passing through a diffraction-limited imaging system can be described as^2,3^

$I\left(x\right)=\int S\left(u\right)\left|t\left(x^{'}\right)h\left(x-x^{'}\right)e^{j2\pi ux^{'}}dx^{'}\right|^{2}du$ (S6)

where $h(x)$ is the point spread function of the imaging system, $S(u)$ represents the intensity distribution in the aperture diaphragm plane, and $t(x)$ is the complex amplitude of the object. This equation indicates that each point on the expanded source illuminates the object from different angles after being collimated by the condenser and produces an image following the linear convolution process. These images are then incoherently superposed with their respective weights, which are defined by the illumination point. Eq. S4 can also be written as

$I\left(x\right)=\iiint S\left(u\right)T\left(u_{1}\right)T^{*}\left(u_{2}\right)H\left(u+u_{1}\right)H^{*}\left(u+u_{2}\right)e^{j2\pi x\left(u_{1}-u_{2}\right)}du_{1}du_{2}du$ (S7)

Due to the superposition process, the image is not linearly related to the object’s complex amplitude but rather to the mixed pairs of its complex amplitude. From Eq. S5, the transmission cross-coefficient (TCC) can be derived as

$TCC(u_{1},u_{2})=\int S\left(u\right)H\left(u+u_{1}\right)H^{*}\left(u+u_{2}\right)du$ (S8)

As demonstrated above, the general imaging formation model is complex and non-linear, which makes the phase recovery particularly challenging. A common strategy to linearize the process is using the weak object approximation, with $a_{0}$ as the mean value of amplitude, and $\alpha$ as the amplitude variation. Under this approximation, the partially coherent image formation model can be written as

(S9)

where $TCC\left(u,0\right)=\int S\left(u'\right)H\left(u'+u\right)H^{*}\left(u'\right)du'$, $\tilde{\alpha}\left(u\right)$ is the Fourier transform of $\alpha$, and $H\left(u\right)$ is the coherent transfer function (CTF). Based on the properties of the Fourier transform, the real function in the spatial domain can be transformed into a combination of a real, even-symmetric function and an imaginary, odd-symmetric function in the frequency domain. Only the imaginary, odd-symmetric part of $TCC(u,0)$ can transfer the complete phase information into the intensity, whereas the real, even-symmetric part can transfer the complete amplitude information. When the imaging system is defocused, the CTF changes to$P\left(u\right)=H\left(u\right)e^{j2\pi\Delta z\sqrt{1/\lambda^{2}-u^{2}}}$. Consequently, the Eq. S7 can be written as

(S10)

where $H_{\alpha}\left(u\right)=2a_{0}\mathrm{Re}\left\{TCC(u,0)\right\}$, and . By computing the intensity difference between two symmetrically defocused images, the amplitude-contributed intensity and the constant background value can be subtracted, yielding a linear equation as

(S11)

The in-focus phase distribution derived from Eq. S11 can be expressed as

(S12)

where .

To improve the deconvolution accuracy described in Eq. S5 and S12, a regulation parameter $\gamma$ is required during calculation, and the Eq. S5 and S12 are converted to:

(S13)

**Supplementary Note 2. Design and imaging performance of a singlet metalens**

The singlet metalens is designed based on the on-axis spherical-aberration-free (OSAF) phase profile (), given by

(S11)

where *r* is the radial coordinate, *s* is the object distance, and *s’* is the image distance. The image magnification is set to 3, and the *NA* is 0.3. The OSAF phase profile demonstrates better on-axis and off-axis imaging performance in microscopy compared to the hyperbolic or quadratic phase^4,5^. Figures S5a, b demonstrate the imaging performance of the OSAF singlet metalens at a wavelength of 450 nm under partially coherent illumination. The partially coherent diffraction limit is defined as ~$\lambda/\left(1+S\right)NA$. As shown in Fig. S5a, when the illumination coherence is high (coherent parameter *S* = 0.17), the ideal half-pitch resolution is 643 nm, and the measured one is between group 9 elements 3 and 4 (half-pitch resolution 690 nm ~ 775 nm). When the illumination coherence is lower (*S* = 0.67), the imaging resolution improves. The ideal half-pitch resolution is 450 nm, and the measured one is between group 10 elements 1 and 2 (half-pitch resolution 435 nm ~ 488 nm, Fig. S5b). Compared to the imaging performance of the optimized doublet given in the manuscript, the on-axis resolution of the OSAF singlet remains the same. However, its near-diffraction-limited FoV is significantly smaller. Severe distortion and blurring due to off-axis aberrations are observed at the edge of the images, with only the region surrounding groups 8 and 9 remaining free of distortion and aberration. This region corresponds to an FoV of 70 μm, which agrees well with the characterization results demonstrated in Fig. 2e of the manuscript. The optical layout, spot diagrams, and MTFs of four object fields from Zemax and the comparisons between the measured Strehl ratios and those from Zemax are presented in Fig. S6.

**Supplementary Note 3. Design of a meta-objective with an imaging magnification of 10 and an *NA* of 0.3**

To demonstrate the potential of our method to achieve a more compact imaging module, we designed a meta-objective with a higher magnification of 10 compared to the meta-objective in the manuscript. When integrated with the image sensor (pixel: 1.67 µm × 1.67 µm, Imaging Source DMM 27UJ003-ML), the objective’s magnification could meet the oversampling requirement to reduce the effect of pixel noise and eliminate the need for an additional relay system. The meta-objective’s design follows the same double-sided scheme with a SiO_2_ substrate thickness of 2.8 mm. Phase profiles of both surfaces follow the expression as (the coefficients $a_{n}$ are shown in Table S3). Figure S11 illustrates the design of the meta-objective working at a wavelength of 450 nm, including its optical layout, spot diagrams, field curvature, distortion, and MTFs calculated in Zemax. The simulation results show a diffraction-limited imaging performance across an FoV of 500 µm with little distortion and field curvature. The total track length is 17.159 mm. The diameters for surface 1 and surface 2 are 0.5 mm and 1.948 mm, respectively. These parameters are compatible with current manufacturing capabilities.


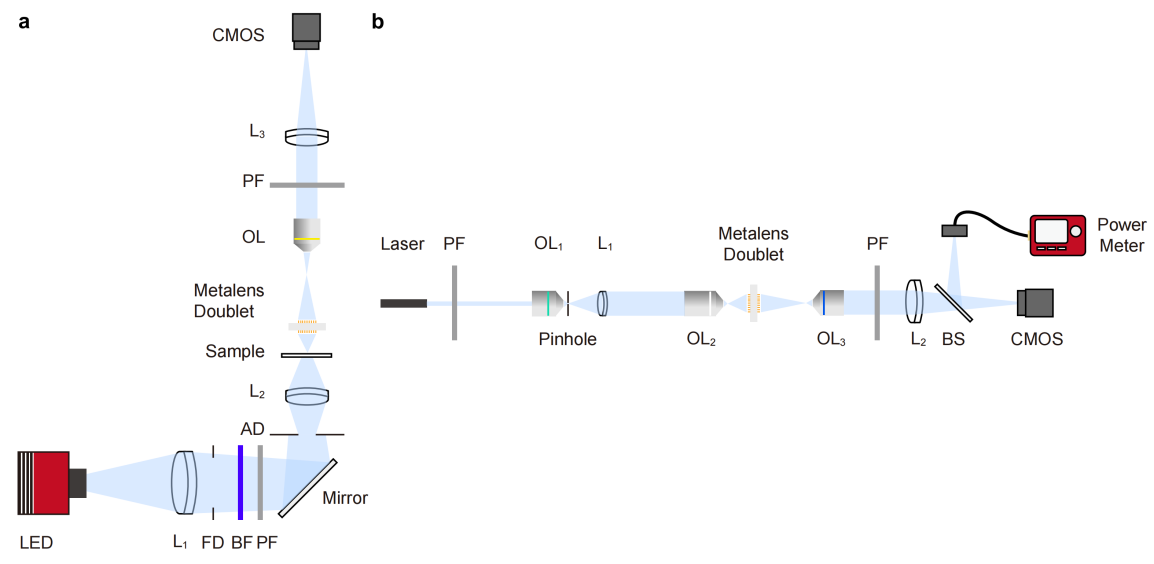


**Fig. S1 Custom-built optical setups for imaging and PSF measurements.** **a** Optical setup for imaging experiments. LED: SOLIS-445C, Thorlabs. Inc. BF: band-pass filter. PF: polarization filter, #88-084, Edmund Optics. FD and AD: field diaphragm and aperture diaphragm, MIS1-25, Jcoptix. OL: NA0.28, 10X, Mitutoyo. CMOS: AIC-2000C-USB, pixel size: 2.4 μm, Jcoptix. L_1_: OLD3254-T2M, Jcoptix. L_2_: OLS240417-T2, Jcoptix. L_3_: TTL200-A, Thorlabs Inc. **b** The optical setup for the PSF and efficiency characterizations. Laser: LDM10-450, Jcoptix. OL_1_: 20X, NA0.4, GCO-2132, Daheng Optics Co. Ltd. OL_2_: LMPLFLN 100X, NA0.8, Olympus. OL_3_: CFI Plan Fluor 40X, NA0.75, Nikon. L_1_: OLD2440-T2M, Jcoptix. L_2_: TTL200-A, Thorlabs Inc. BS: 50-50 beam splitter, OSB25R55-T2, Jcoptix. Power meter: PM120D, Thorlabs Inc.


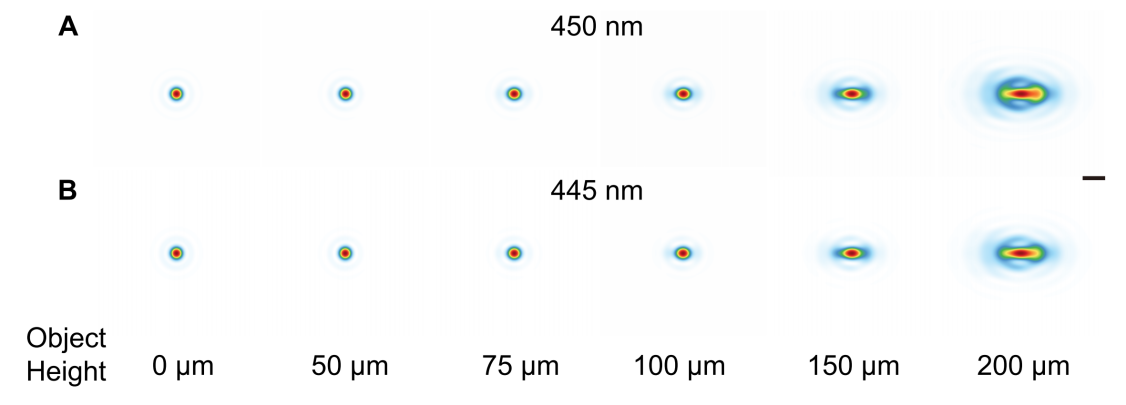


**Fig. S2 Simulated PSFs of the meta-objective at wavelengths of 445 nm and 450 nm. a** Simulated PSFs of the meta-objective with a point source located at different object heights at a wavelength of 450 nm. **b** Simulated PSFs at a wavelength of 445 nm. The designed image distance is ~3.667 mm, with corresponding object distances of ~725 μm and ~735 μm accordingly. The imaging magnifications are 4 and 3.977, respectively. Scale bar: 4 μm.


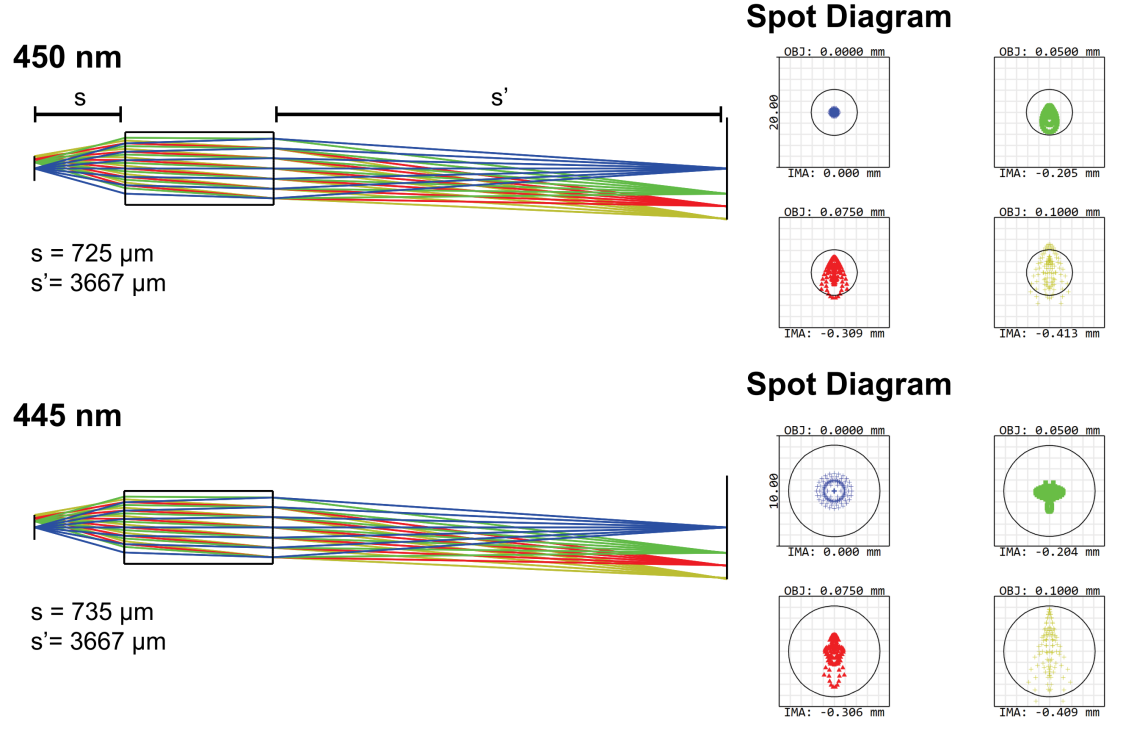


**Fig. S3.** Demonstration of the meta-objective layouts for four object fields (object heights: 0 μm, 50 μm, 75 μm, and 100 μm) at wavelengths of 450 nm and 445 nm, and the corresponding spot diagrams in the image plane.


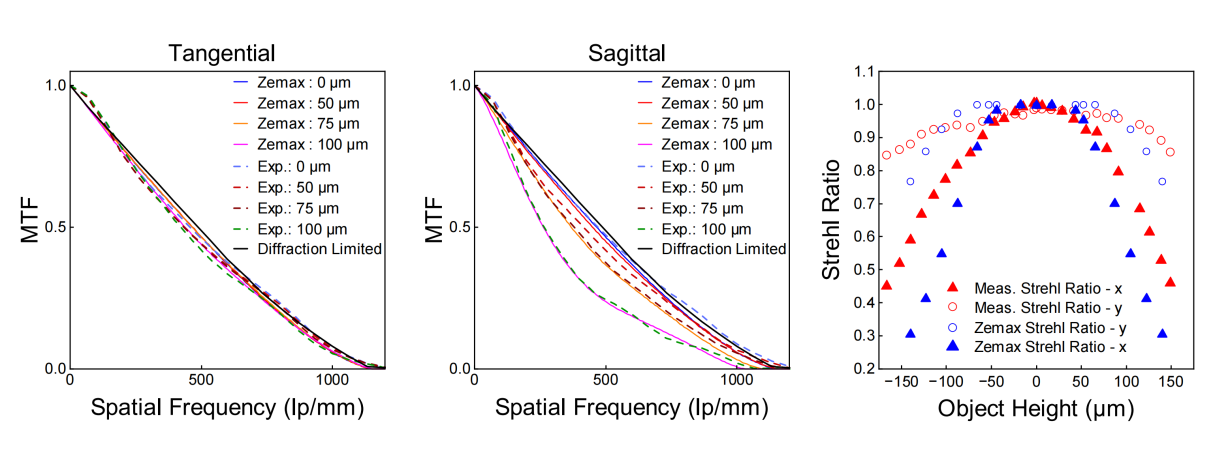


**Fig. S4** **Comparison between measured MTFs and Strehl ratios with those from Zemax.** The measured data is similar to the results from Zemax, indicating good performance.


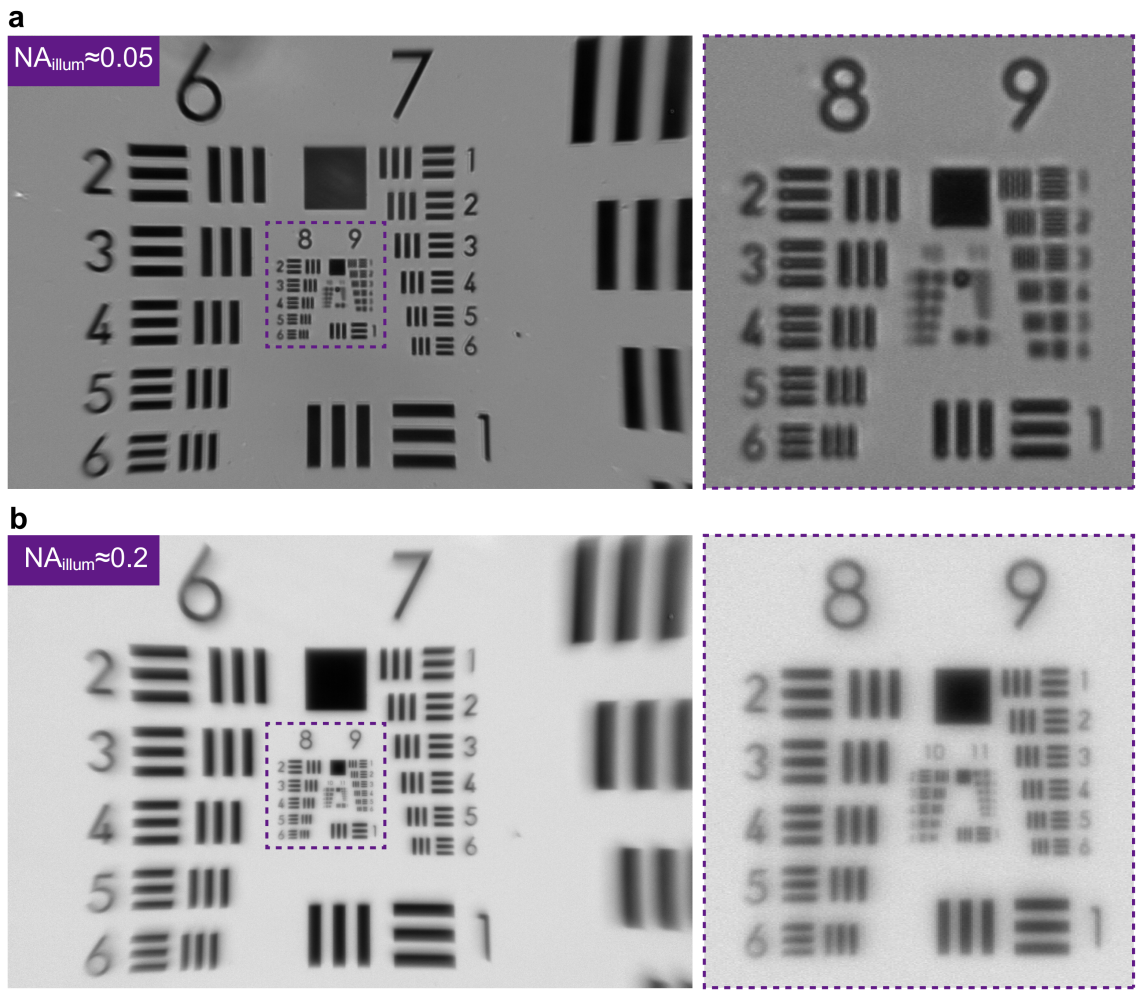


**Fig. S5 Images of the OSAF singlet metalens captured by the relay system.** **a** Resolution test chart image acquired under low *NA* illumination, where *S* = 0.17. **b** Resolution test chart image acquired under high *NA* illumination, where *S* = 0.67. All images are flat-field corrected. The width of the image is 439 μm and the height is 294 μm.


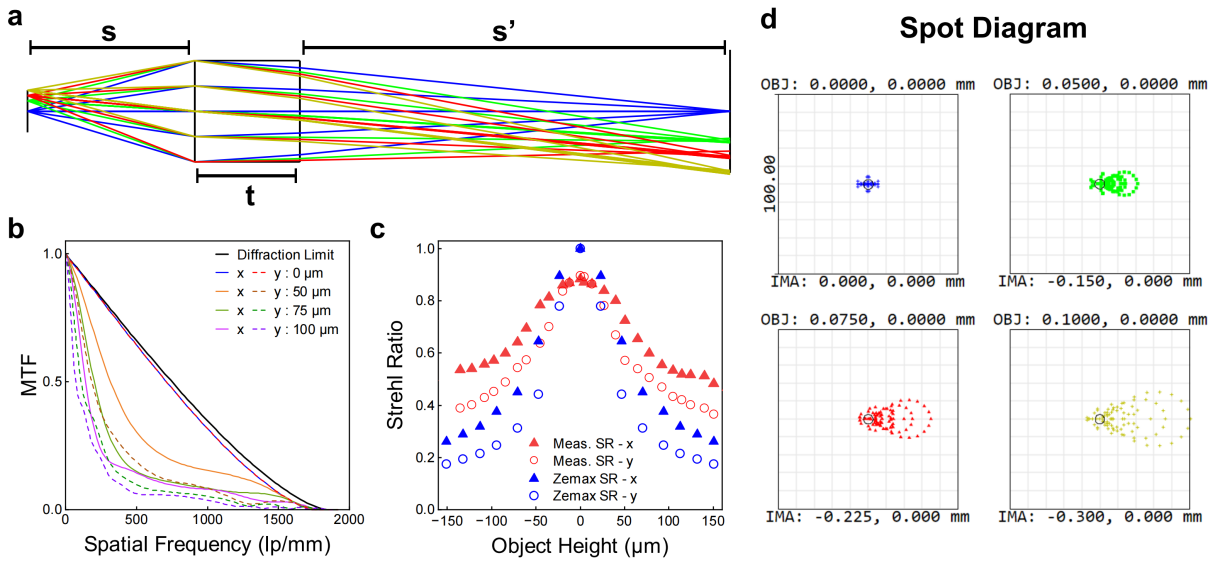


**Fig. S6 Zemax simulation analysis of the OSAF metalens a** Optical layout of four object fields (object heights: 0 μm, 50 μm, 75 μm, and 100 μm) at a wavelengths of 450 nm. The lens diameter is 0.5 mm. The object distance *s* = 0.795 mm, the image distance *s’* = 2.047 mm, and the thickness of the SiO_2_ substrate *t* = 0.5 mm. **b** MTFs along x and y directions from Zemax. **c** Comparison between the measured Strehl ratios of the OSAF metalens and those from Zemax. **d** Spot diagrams in the image plane of the four object fields.


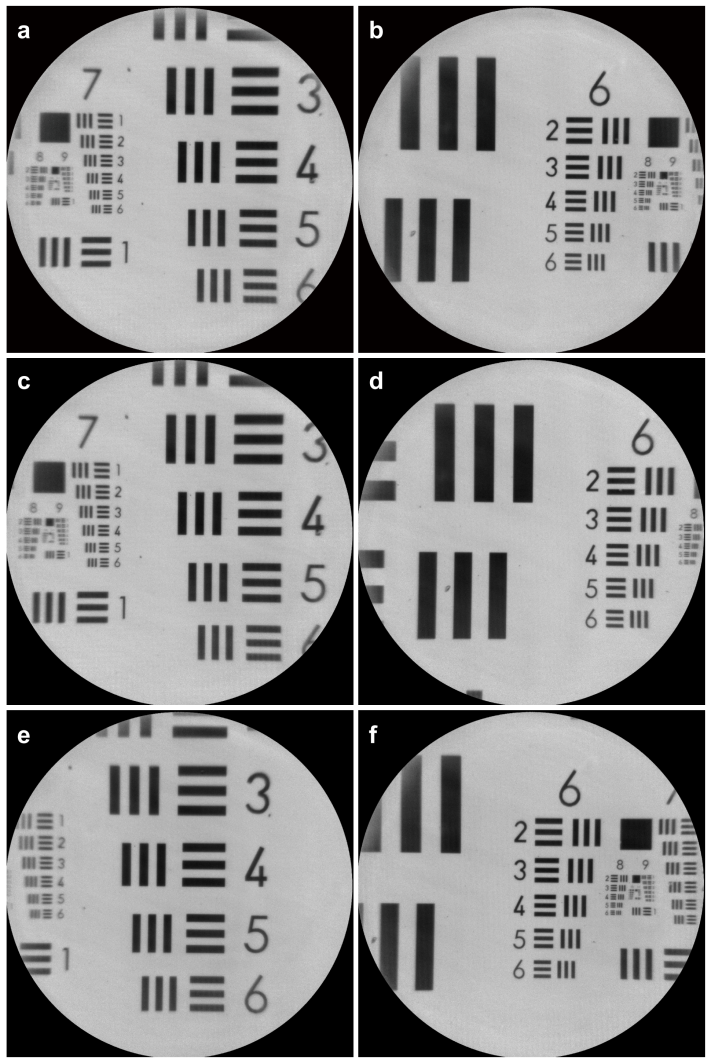


**Fig. S7 Flat-field corrected images of the meta-objective captured directly by the imaging sensor at a magnification of 4.** **a**, **b** These images demonstrate positions where elements in group 9 can no longer be distinguished, indicating FoV of ~313 μm. **c**, **d** These two images demonstrate that group 8 element 3 (half-pitch resolution of 1.55 μm) can still be distinguished at the edge of the entire FoV of 440 μm. **e**, **f** These two images demonstrate that all elements in group 7 can be clearly distinguished at the entire FoV of 440 μm.


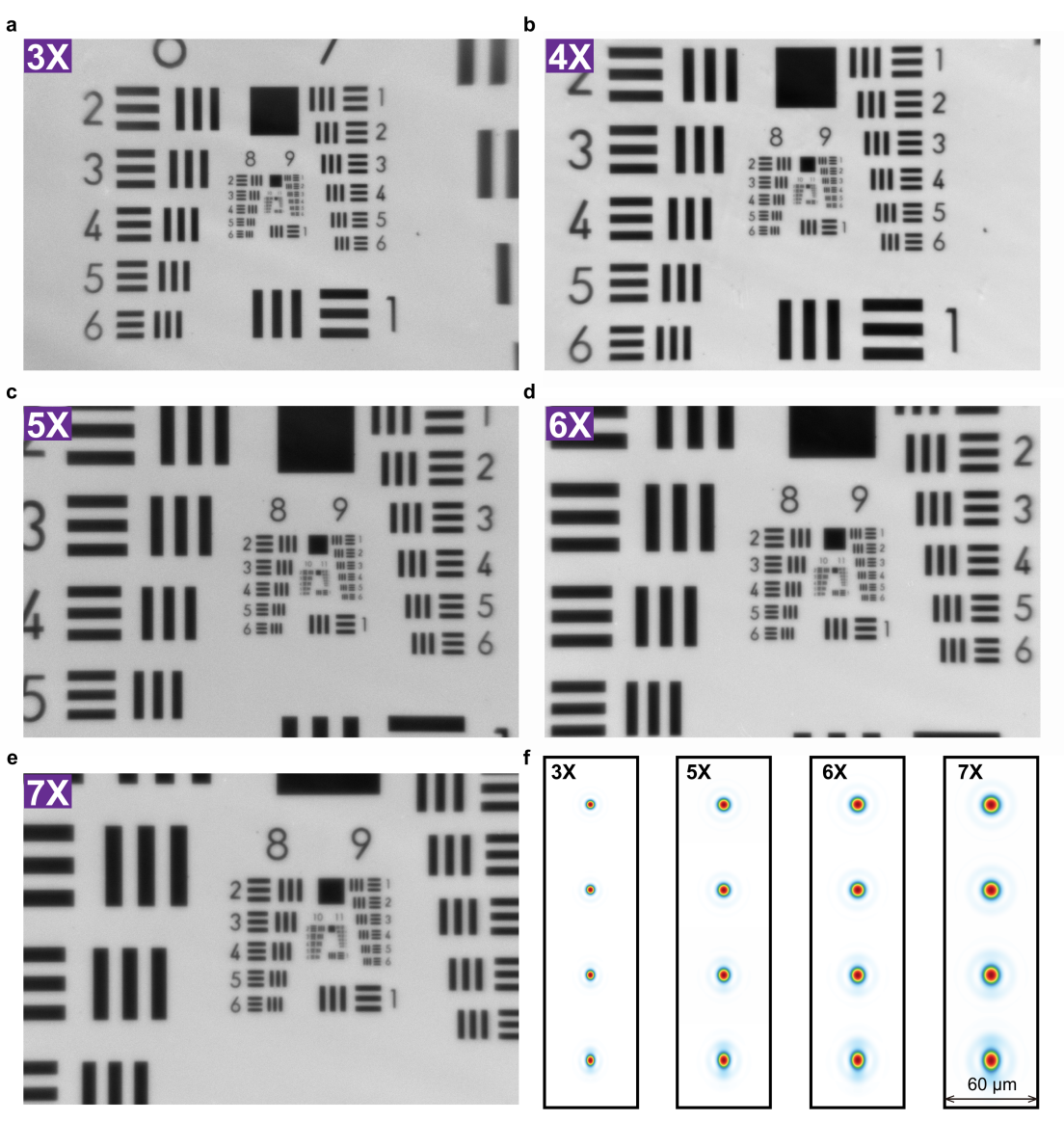


**Fig. S8 Images of the meta-objective captured after the relay system.** **a**-**e** Resolution test chart images captured with the meta-objective under varying magnifications. All images are flat-field corrected and demonstrate diffraction-limited imaging performance within a 200-μm FoV. **f** Simulated PSFs under different imaging magnifications. The respective object heights are 0 μm, 50 μm, 75 μm, and 100 μm from top to bottom. The PSFs with different magnifications display similar shapes, and no obvious deteriorations exist, meaning the near-diffraction-limited FoV remains at 200 μm till the magnification of 7. The working wavelength is 450 nm.


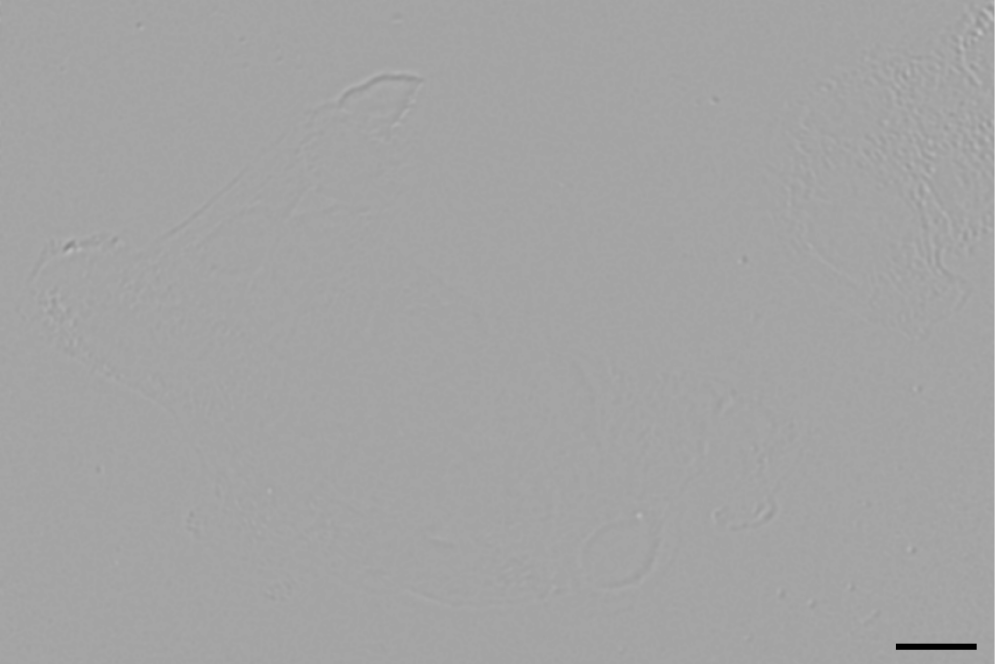


**Fig. S9 In-focus intensity image of the HeLa cells**. The intensity image shows little contrast of the cell morphology. The image is flat-field corrected, and the working wavelength is 450 nm. Scale bar: 20 μm.


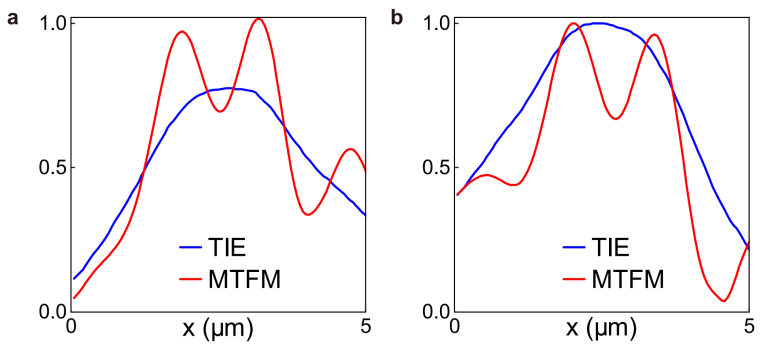


**Fig. S10 Comparison of edge profiles obtained using the MTFM and TIE method along the yellow arrows in Figs. 5e and 5h of the manuscript.** **a** The yellow arrow in Fig. 5e in the manuscript marks the border between the cell and the ambient medium. The MTFM-based edge profiles reveal two distinct edges: one at the interface between the nucleus and the cytoplasm, and another at the interface between the cytoplasm and the ambient medium. These features are absent in the TIE-based profile. **b** A similar effect is observed, further demonstrating the superior edge detection capability of the MTFM compared to the TIE method.


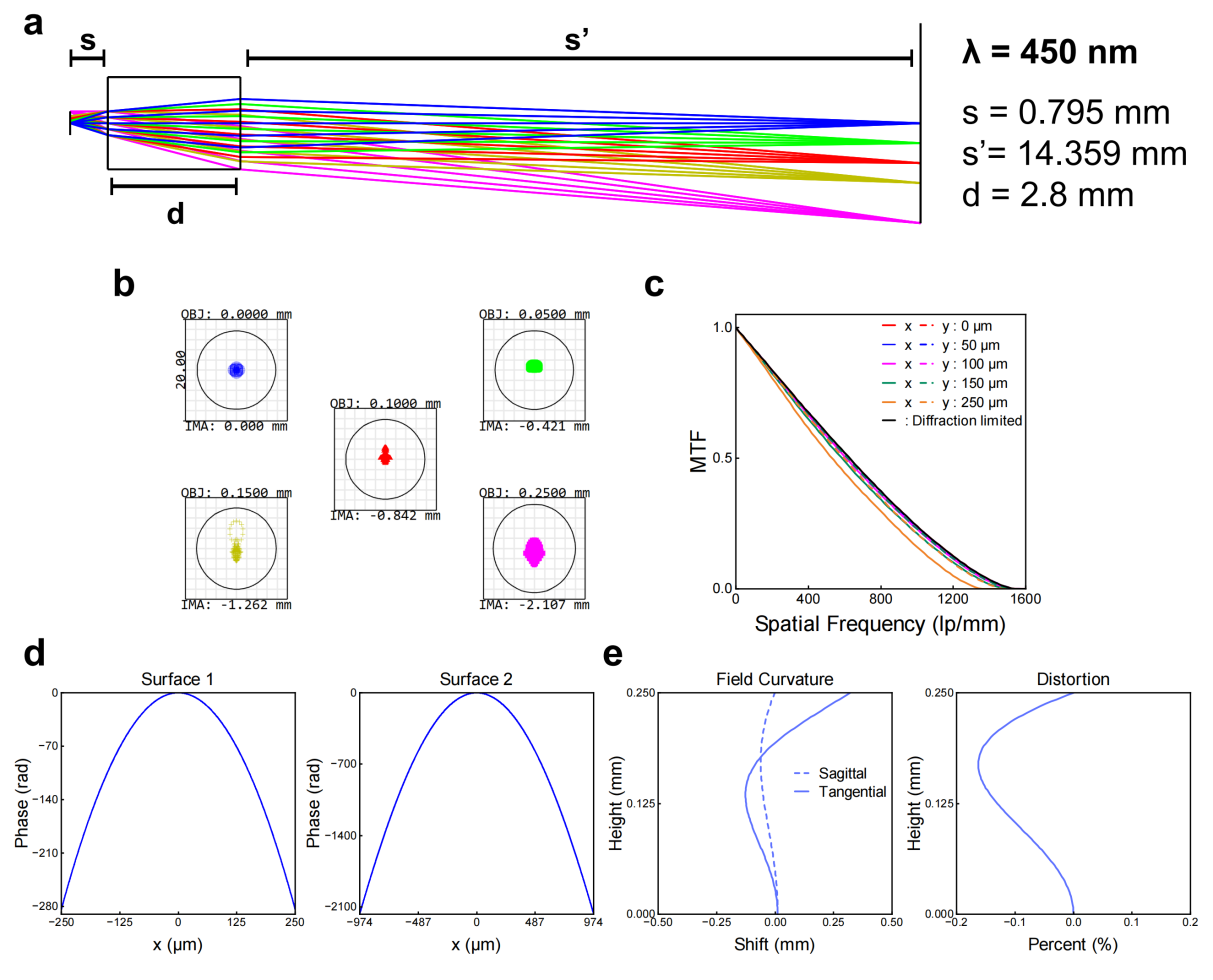


**Fig. S11 Demonstration the Zemax simulation results of the meta-objective with an *NA* of 0.3 and a magnification of 10. a** Optical layout of the meta-objective at a wavelength of 450 nm. **b** Spot diagrams in the image plane of the five object fields (0 µm, 50 µm, 100 µm, 150 µm, and 250 µm), all constrained within the Airy disk. **c** MTFs of the five fields. **d** Phase profiles of the surface 1 and 2 of the meta-objective. **e** The field curvature and distortion of the meta-objective.

**Table S1 QPI performance of this work and the state of the art.**

| **Reference** | **Isotropic**  **(Yes or No)** | **Half-pitch resolution (μm)** | **FoV optimized**  **(Yes or No)** |
| --- | --- | --- | --- |
| Ref. 27 | No | Horizontal: 2.19  Longitudinal: 2.76 | No |
| Ref. 28 | No | Horizontal: 1.38  Longitudinal: 1.74 | Yes |
| Ref. 29 | Yes | 18.52* | No |
| Ref. 30 | Yes | 2.46 | No |
| Ref. 31 | Yes | 1.09* | No |
| Ref. 32 | Yes | 6.23* | No |
| Ref. 33 | Yes | 0.56 | No |
| Ref. 34 | Yes | 5.41* | No |
| **This work** | **Yes** | **0.488** | Yes |

*The lateral resolution is not characterized in the work, and the resolution shown here is approximated using the Rayleigh criterion, 0.61*λ*/*NA*, where *λ* is the working wavelength, *NA* is the numerical aperture of the lens at the object space, *s* is the object distance, and *R* is the radius of the lens.

**Table S2 Coefficients of the phase profiles of the plan meta-objective.**


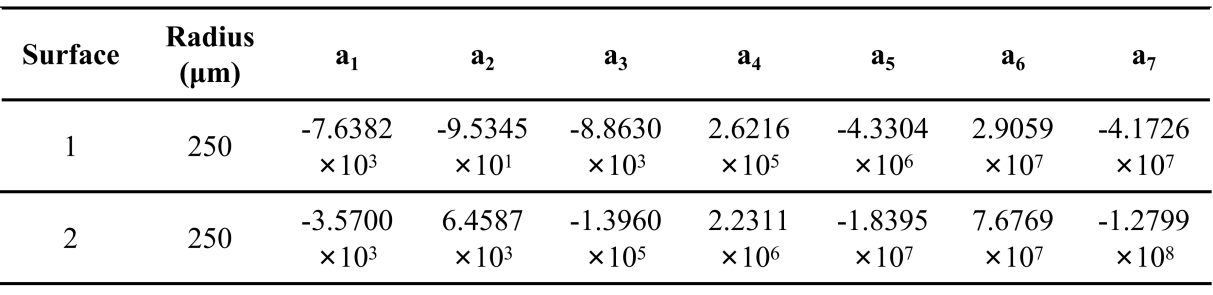


The designed image distance is 3.667 mm, with corresponding object distances of 725 μm at a wavelength of 450 nm and 735 μm at a wavelength of 445 nm. The imaging magnifications are 4 and 3.977, respectively. The effective focal length of the meta-objective is 876 μm. The focal length of surface 1 is 924 μm, and that of surface 2 is 1978 μm.

**Table S3 Coefficients of the phase profiles of the meta-objective with an *NA* of 0.3 and a magnification of 10.**


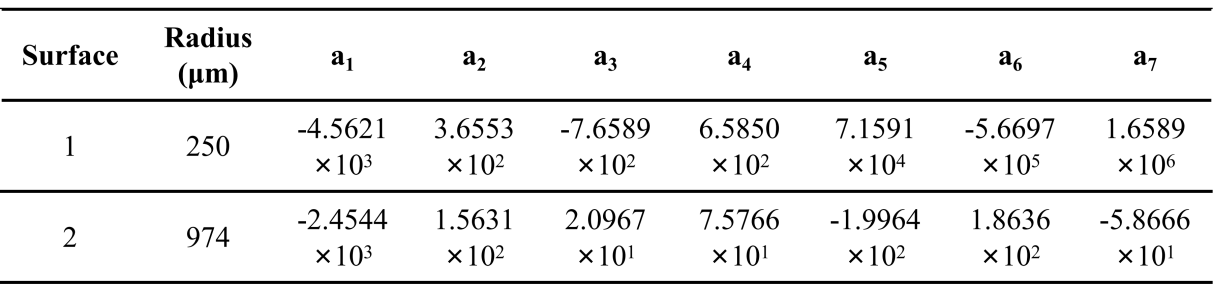


**Reference**

1. Teague, M. R. Deterministic phase retrieval: a Green’s function solution. *Journal of the Optical Society of America* **73**, 1434–1441 (1983).

2. Zuo, C. et al*.* Transport of intensity equation: a tutorial. *Optics and Lasers in Engineering* **135**, 106187 (2020).

3. Zuo, C. et al. Transport of intensity phase retrieval and computational imaging for partially coherent fields: The phase space perspective. *Optics and Lasers in Engineering* **71**, 20–32 (2015).

4. Chen, C. et al*.* Bifacial-metasurface-enabled pancake metalens with polarized space folding. *Optica* **9**, 1314-1322 (2022).

5. Ye, X. et al. Ultracompact Multimode Meta-Microscope Based on Both Spatial and Guided-Wave Illumination. Advanced Devices & Instrumentation **4**:0023 (2023).
